# Readmission outcomes following infectious hospitalization: same-care unit performed better than different-care unit

**DOI:** 10.1186/s12913-023-09220-1

**Published:** 2023-03-10

**Authors:** Yi-Chin Pu, Hsiao-Chen Chou, Chun-Ta Huang, Wang-Huei Sheng

**Affiliations:** 1 Department of Nursing, National Taiwan University Hospital, Taipei, Taiwan; 2 Department of Internal Medicine, National Taiwan University Hospital, No. 7, Chung-Shan South Road, Taipei, Taiwan; 3 Graduate Institute of Clinical Medicine, National Taiwan University, Taipei, Taiwan

**Keywords:** Continuity of care, Infection, Length of stay, Mortality, Readmission

## Abstract

**Background:**

Previous studies showed that same-hospital readmission is associated with better outcomes than different-hospital readmission. However, little is known about whether readmission to the same care unit (same-care unit readmission) after infectious hospitalization performs better than readmission to a different care unit at the same hospital (different-care unit readmission).

**Methods:**

This retrospective study screened patients rehospitalized within 30 days following admission to two acute medical wards for infectious diseases from 2013 to 2015 and included only those readmitted for unplanned medical reasons. Outcomes of interest included hospital mortality and length of stay of readmitted patients.

**Results:**

Three hundred and fifteen patients were included; of those, 149(47%) and 166(53%) were classified as same-care unit and different-care unit readmissions, respectively. Same-care unit patients were more likely to be older(76 years vs. 70 years; P = 0.001), have comorbid chronic kidney disease(20% vs. 9%; P = 0.008), and have a shorter time to readmission(13 days vs. 16 days; P = 0.020) than different-care unit patients. Univariate analysis showed that same-care unit patients had a shorter length of stay than different-care unit patients(13 days vs. 18 days; P = 0.001), but had similar hospital mortality(20% vs. 24%; P = 0.385). The multivariable linear regression model indicated that same-care unit readmission was associated with a 5-day shorter hospital stay than different-care unit readmission(P = 0.002).

**Conclusion:**

Among patients readmitted within 30 days after hospitalization for infectious diseases, same-care unit readmission was associated with a shorter length of hospital stay than different-care unit readmission. Whenever feasible, it is encouraged to allocate a readmitted patient to the same care unit in hope of pursuing continuity and quality of care.

## Introduction

Infectious diseases are one of the leading causes of admissions and deaths around the globe.[1] Following an infectious admission, a significant proportion of patients will experience readmissions for a variety of reasons.[2] Hence, it is of paramount importance to understand how to improve the outcomes of readmitted patients. Prior studies have shown that a readmission to the same hospital following an index admission for heart failure,[3] acute pancreatitis,[4] acute stroke,[5] transcatheter aortic valve implantation,[6] or cirrhosis[7] is associated with a better outcome than a readmission to a different hospital. However, little, if any, is known about whether patients readmitted to the same care unit after an infectious hospitalization will have a better prognosis than those readmitted to a different care unit of the same hospital. Thus, in this study, we aimed to investigate the effects of medical care provided in the same care unit on the outcome of readmitted patients after a prior admission for infections.

## Methods

### Study settings and participants

This retrospective study was conducted at the National Taiwan University Hospital, a tertiary-care referral medical center in North Taiwan. From 2013 to 2015, all patients admitted to the two acute medical wards were screened for eligibility. The included criteria were as follows: (a) main admission diagnosis of infectious diseases; (b) survival to hospital discharge; and (c) readmitted to the same hospital within 30 days following discharge. Patients were excluded if they (a) were discharged against medical advice; (b) were deemed to be imminently dying and were discharged for palliative care; (c) had a hospital admission within 1 year prior to the index admission; (d) were readmitted for scheduled procedures, surgeries, or medical therapies; (e) had a non-medical readmission diagnosis, such as fractures and burn injuries; and (f) were readmitted to the intensive care units.

During the study period, the two acute medical wards, equipped with 71 beds, mainly accommodated patients from the emergency department and were run by a fixed health care provider team, comprising 8 attending physicians with specialties in renal, respiratory, infectious, and gastrointestinal disease and family medicine. The settings of wards in our hospital were basically the same and Taiwan had run a National Health Insurance program in which all citizens were enrolled irrespective of their financial status. Therefore, a significant difference in medical expenses between hospitalizations to different wards was not expected to happen. The assignments of the health care providers across wards were fixed to a certain extent and the vast majority of attending physicians in our institution were board-certified specialists. Ethics approval was obtained from the Research Ethics Committee of the National Taiwan University Hospital and informed consent was waived given the retrospective nature of the study.

### Data collection and outcomes of interest

Patient records were reviewed in detail to retrieve the information of the index admission as follows: age, gender, comorbidities, sites of infections, pertinent clinical events (septic shock, acute kidney injury, and the use of mechanical ventilation), and length of stay. The Charlson Comorbidity Index was calculated to assess comorbidity burden at the time of the index admission.[8] Sites of infections were classified according to the Centers for Disease Control and Prevention definitions.[9] For readmission parameters, the data items captured were time to readmission, main readmission diagnosis, length of stay, and hospital mortality. The principal diagnoses of readmission were further categorized as infectious versus non-infectious for analysis.

The primary outcome of interest was hospital mortality during the readmission. Another outcome of interest was the length of stay of the readmission. The main objective was to compare outcomes for readmission to the same care unit following the index admission versus readmission to a different care unit at the same hospital.

### Statistical analysis

Statistical software, SPSS, version 19.0 (SPSS Inc., Chicago, Illinois, US) was used to compute descriptive and predictive statistics. A P value of < 0.05 was deemed statistically significant. The dataset was divided into two groups: those for whom the readmission was to the same care unit and those who were readmitted to a different care unit. Comparisons between the two groups were then conducted for the continuous variables using an independent sample t-test, whereas Pearson’s Chi-squared test was used for comparisons of the categorical variables. In multivariable regression models, we examined two main readmission outcomes: length of stay in days (using linear regression) and hospital mortality (using logistic regression). For linear regression models, we reported differences in length of stay (and their 95% confidence intervals [CIs]) for the main predictor, and for logistic regression models, we reported the odds ratios (ORs) (and their 95% CIs) for hospital mortality.

We ran 3 sets of models: (1) adjusting for patient demographics and comorbidities; (2) adjusting for patient demographics, comorbidities, and variables (clinical events and length of stay) at the time of the index admission; and (3) adjusting for patient demographics, comorbidities, variables (clinical events and length of stay) at the time of the index admission, and variables (time to readmission and readmission diagnosis) at the time of the readmission, in order to control for confounders that may interfere with the outcomes of the readmitted patients.

## Results

**Fig. 1:**
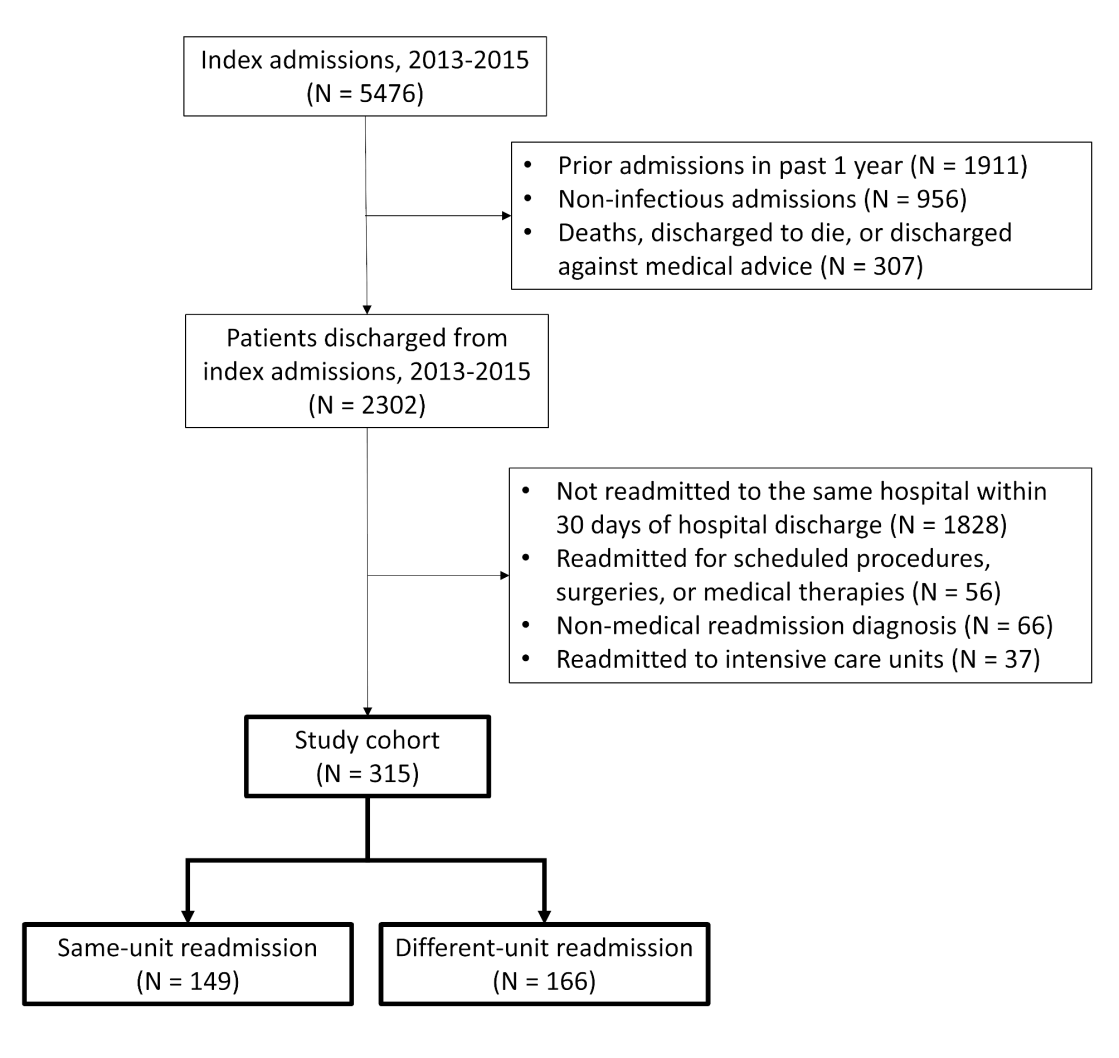
Study flow diagram

During the study period, a total of 315 readmission patients were included (Fig. 1). Of those, 149 (47%) and 166 (53%) were readmitted to the same care unit (same-care unit readmission) and a different care unit (different-care unit readmission), respectively (Table 1). The average age of the study population was 73 years and the sex ratio was approximately 1:1. The most common comorbidities were malignancy (43%), diabetes mellitus (33%), and cerebrovascular disease (22%). Pneumonia (63%) and urinary tract infection (28%) accounted for the majority of the index infectious admissions. There were 44 (14%), 26 (8.3%), and 12 (3.8%) patients experiencing acute kidney injury, septic shock, and the use of mechanical ventilation during the index admission. The mean length of stay of the index admission was 11 days.

**Table 1:** Patient characteristics

Variable	Different-unit readmission (N = 166)	Same-unit readmission (N = 149)	P value
Age, years	70 ± 16	76 ± 13	0.001
≥65	114 (69)	118 (79)	0.034
Male gender	89 (54)	83 (56)	0.710
Charlson comorbidity index	2.8 ± 2.3	2.7 ± 2.3	0.574
Comorbidity			
Malignancy	73 (44)	61 (41)	0.586
Diabetes mellitus	60 (36)	43 (29)	0.169
Cerebrovascular disease	31 (19)	37 (25)	0.185
Coronary artery disease	25 (15)	21 (14)	0.808
Chronic kidney disease	15 (9.0)	29 (20)	0.008
Heart failure	14 (8.4)	17 (11)	0.376
Chronic obstructive pulmonary disease	10 (6.0)	14 (9.4)	0.260
Liver cirrhosis	6 (3.6)	4 (2.7)	0.754
Site of infection during index admission			
Pneumonia	99 (60)	98 (66)	0.262
Intraabdominal infection	24 (15)	13 (8.7)	0.115
Skin and soft tissue infection	6 (3.6)	10 (6.7)	0.211
Urinary tract infection	47 (28)	42 (28)	0.980
Primary bacteremia	5 (3.0)	7 (4.7)	0.435
Febrile neutropenia	7 (4.2)	6 (4.0)	0.933
Others	7 (4.2)	7 (4.7)	0.836
Clinical event during index admission			
Intensive care unit care	9 (5.4)	8 (5.4)	0.984
Septic shock	13 (7.8)	13 (8.7)	0.774
Acute kidney injury	21 (13)	23 (15)	0.476
Mechanical ventilation	6 (3.6)	6 (4.0)	0.849
Length of stay, index admission, days	11 ± 10	12 ± 8	0.156

Mean ± SD is shown for continuous variables, and the numbers in brackets for categorical variables indicate %

Same-care unit patients were more likely to be older (76 years vs. 70 years; P = 0.001) and have comorbid chronic kidney disease (20% vs. 9.0%; P = 0.008) than different-care unit patients. Same-care unit patients also had a shorter time to readmission (13 days vs. 16 days; P = 0.020) and were more likely to be readmitted with an infectious diagnosis (79% vs. 67%; P = 0.021) than different-care unit patients (Table 2). More than two thirds (72%) of all the readmissions could be ascribed to infectious diseases. Of those, pneumonia (40%), urinary tract infection (13%), and intraabdominal infection (8.8%) were the most common encounters (Table 2).

**Table 2:** Readmission outcomes, time to readmission, and readmission diagnosis

Variable	Different-unit readmission (N = 166)	Same-unit readmission (N = 149)	P value
Time to readmission, days	16 ± 8	13 ± 8	0.020
Readmission diagnosis			
Infectious disease	111 (67)	117 (79)	0.021
Pneumonia	57 (34)	68 (46)	
Urinary tract infection	23 (14)	17 (11)	
Intraabdominal infection	17 (10)	11 (7.4)	
Skin and soft tissue infection	5 (3.0)	5 (3.4)	
Catheter-related bloodstream infection	2 (1.2)	4 (2.7)	
Primary bacteremia	1 (0.6)	2 (1.3)	
Others	6 (3.6)	10 (6.7)	
Non-infectious disease	55 (33)	32 (22)	
Gastrointestinal	13 (7.8)	14 (9.4)	
Cardiovascular	11 (6.6)	3 (2.0)	
Respiratory	10 (6.0)	3 (2.0)	
Neurologic	10 (6.0)	2 (1.3)	
Urinary	2 (1.2)	4 (2.7)	
Hepatobiliary	3 (1.8)	2 (1.3)	
Others	6 (3.6)	4 (2.7)	
Outcome during readmission			
Mortality	39 (24)	29 (20)	0.385
Infectious cause	19(11)	14(9.4)	
Non-infectious cause	20(12)	15(10)	
Length of stay, days	18 ± 18	13 ± 10	0.001

Mean ± SD is shown for continuous variables, and the numbers in brackets for categorical variables indicate %

The unadjusted outcome model showed that same-care unit patients had a significantly shorter length of stay than different-care unit patients (13 days vs. 18 days; P = 0.001) but had a similar hospital mortality rate (20% vs. 24%; P = 0.385). On the multivariable linear regression model adjusting for age, sex, and comorbidity (model 1), same-care unit readmission was associated with a 5-day shorter hospital stay than different-care unit readmission (P = 0.002). A similar finding was found in both adjusted models 2 and 3 (Table 3).

**Table 3:** Impact of same-care unit vs. different-care unit readmission on length of stay and hospital mortality

Same-unit readmission vs. Different-unit readmission	Length of stay, days			Hospital mortality, OR		
	Difference	95% CI	P value	Estimate	95% CI	P value
Unadjusted model	-5.2	-8.4 ~ -2.0	0.002	0.787	0.458 ~ 1.352	0.385
Adjusted model						
Model 1^a^	-5.3	-8.7 ~ -1.9	0.002	0.885	0.499 ~ 1.571	0.608
Model 2^b^	-4.9	-8.4 ~ -1.6	0.004	0.834	0.467 ~ 1.490	0.539
Model 3^c^	-5.5	-8.9 ~ -2.2	0.001	0.895	0.496 ~ 1.617	0.713

CI, confidence interval; OR, odds ratio

^a^ Adjusted for age, sex, and comorbidity

^b^ Adjusted for age, sex, comorbidity, and clinical event and length of stay during index admission

^c^ Adjusted for age, sex, comorbidity, clinical event and length of stay during index admission, time to readmission, and readmission diagnosis

## Discussion

To the best of our knowledge, this study was the first one to investigate the impact of same-care unit readmission versus different-care unit readmission at the same hospital after an index admission for infectious diseases on patient outcomes in terms of length of stay and hospital mortality during the readmission. The main findings were as follows: (a) patients with an older age, comorbid chronic kidney disease, a shorter time to readmission, and a principal readmission diagnosis of infections were more likely to be admitted to the same care unit; (b) same-care unit readmission was associated with a shorter length of stay of than different-care unit readmission; and (c) hospital mortality was similar regardless of the unit of readmission. In short, the results of the present study suggest that, where possible, patients with a medical admission diagnosis should be readmitted to the same care unit following an index infectious hospitalization for continuity of care and better prognosis of the patients.

Unplanned readmissions to the hospital within 30 days of discharge from medical wards occur in up to 20% of adults and the readmission rate is widely considered a quality indicator for hospital care.[10, 11] Therefore, emphasis has been placed to identify causal risk factors or high-risk patient groups amenable to interventions to reduce readmissions.[12–18] However, not all of the readmissions are avoidable.[19] When readmissions are deemed unavoidable, same-hospital readmission has been one of the measures to improve patient outcomes, i.e., length of stay or mortality, following hospitalizations for several medical conditions.[3–7] Proposed advantages of same-hospital readmission include ready access to health information, decreased barriers to communication between health care providers, and diminution in duplicated laboratory and radiological investigations.[20–23] The work carried out in this study added to existing knowledge by showing that beyond same-hospital admission, same-care unit readmission after an index infectious hospitalization was beneficial in terms of hospital length of stay. This observation may be explained by a better rapport between the patient, family, and health care team, and a better understanding of their needs. In this regard, patient care may be more efficiently delivered and a discharge plan can be more easily tailored to suit the patient’s specific needs.

Our major outcome of interest was the hospital mortality of patients requiring a readmission. We sought to identify whether readmission to a different care unit other than the same care unit impacted the outcome. Our model showed that different-care unit readmission did not increase the risk of mortality in patients following infectious hospitalizations. The finding may not be unexpected because of shared medical records, facilities, and resources for specialty consultation in both settings of a single institution. Another explanation for this finding is that the health care providers will not be clouded in clinical judgment due to previous experience with treating the patient, which may lead to fewer missed diagnoses and medical errors. Thus, the hard outcome measure of the patients could not have been affected by allocation of patient care.

In this study, we also explored the clinical features associated with same- or different-care unit readmission, and found that patients with an older age, comorbid chronic kidney disease, a shorter time to readmission, and an infectious readmission diagnosis were more likely to be allocated to the same care unit. In fact, there is no structured rule for allocating a patient who requires inpatient care in our hospital. This means that the bed managers may take these patient-level factors into account while placing patients in beds, although we were not able to access their decision-making process. The association between same-care unit readmission and a shorter length of hospital stay observed in this work suggests that the continuity of care should be prioritized in bed management even within a single institutional setting.

Pneumonia accounted for approximately two-thirds of the index admissions in the present work. Prior studies have demonstrated that destabilization of comorbidities, such as chronic obstructive pulmonary disease and heart failure, was the main reason for readmission after a hospitalization for pneumonia.[24, 25] In contrast to those studies, infectious diseases were the major readmission diagnoses in our study cohort. The discrepancies may be best ascribed to the differences in the patient population, i.e., a higher proportion of comorbid malignancy (43% vs. 10–19%) and a lower proportion of comorbid chronic obstructive pulmonary disease (8% vs. 33–61%) and heart failure (10% vs. 21–41%) for our patients compared to those patients in previous studies.[24, 25] In this regard, a precaution should be taken while applying our study findings to another patient population with different comorbidity profiles and reasons for rehospitalization.

A number of limitations to this study should be mentioned. First, our findings were based on a single-center experience and may not be generalizable to other health care settings, such as regional hospitals and district hospitals. However, as a pioneer study in this field, our promising results will encourage more future studies to validate our observations. Second, we only included patients readmitted after an infectious hospitalization since infections were the leading admission diagnoses for the two acute medical wards. Thus, it remains to be evaluated whether our conclusion can be extrapolated to other medical diseases. Nonetheless, we believe that the benefits of continuity of health care can be observed across different disease entities.[26] Third, our results should be interpreted with caution because some unmeasured confounders could exist in a retrospective study. Nevertheless, a real prospective randomized controlled trial may never be done since bed management in the hospital is a complicated process that involves bed availability, patient preferences, hospital policies, among others. Fourth, in order to assemble a well-defined patient population, a significant number of patients with prior healthcare exposure within the past year were excluded because of diverse backgrounds in terms of the diagnosis, intervention, complication, and number of previous admissions. This may limit the generalizability of our study findings.

In conclusion, same-care unit readmission was associated with a significantly shorter length of stay than different-care unit readmission after an infectious hospitalization, although hospital mortality did not differ between two readmission settings. The findings of this study suggest that, whenever feasible, it is encouraged to allocate a readmitted patient to the same care unit in hope of pursuing continuity and quality of patient care.

## Data Availability

The datasets used and/or analyzed during the current study are available from the corresponding author on reasonable request.
